# Intraspecific variation in root and leaf traits and leaf-root trait linkages in eight aspen demes (*Populus tremula* and *P. tremuloides*)

**DOI:** 10.3389/fpls.2013.00415

**Published:** 2013-10-21

**Authors:** Peter Hajek, Dietrich Hertel, Christoph Leuschner

**Affiliations:** Plant Ecology and Ecosystems Research, Albrecht von Haller Institute for Plant Sciences, University of GöttingenGöttingen, Germany

**Keywords:** fine root morphology, genetic variation, intraspecific variation, relative growth rate, root tissue density, specific root area

## Abstract

Leaf and fine root morphology and physiology have been found to vary considerably among tree species, but not much is known about intraspecific variation in root traits and their relatedness to leaf traits. Various aspen progenies (*Populus tremula* and *P. tremuloides*) with different growth performance are used in short-rotation forestry. Hence, a better understanding of the link between root trait syndromes and the adaptation of a deme to a particular environment is essential in order to improve the match between planted varieties and their growth conditions. We examined the between-deme (genetic) and within-deme (mostly environmental) variation in important fine root traits [mean root diameter, specific root area (SRA) and specific root length (SRL), root tissue density (RTD), root tip abundance, root N concentration] and their co-variation with leaf traits [specific leaf area (SLA), leaf size, leaf N concentration] in eight genetically distinct *P. tremula* and *P. tremuloides* demes. Five of the six root traits varied significantly between the demes with largest genotypic variation in root tip abundance and lowest in mean root diameter and RTD (no significant difference). Within-deme variation in root morphology was as large as between-deme variation suggesting a relatively low genetic control. Significant relationships existed neither between SLA and SRA nor between leaf N and root N concentration in a plant. Contrary to expectation, high aboveground relative growth rates (RGR) were associated with large, and not small, fine root diameters with low SRA and SRL. Compared to leaf traits, the influence of root traits on RGR was generally low. We conclude that aspen exhibits large intraspecific variation in leaf and also in root morphological traits which is only partly explained by genetic distances. A root order-related analysis might give deeper insights into intraspecific root trait variation.

## Introduction

Leaf morphology and foliar nitrogen (N) content are easy to measure plant traits that have widely been used for characterizing plant growth and resource use strategies (e.g., Reich et al., 1997; Diaz et al., 2004). The analysis of large data bases has revealed general patterns of leaf trait syndromes (e.g., Reich et al., 2003) which reflect trade-offs in terms of energy requirements (Wright et al., 2004) and physical constraints of plant growth. Much less information exists about root traits, in particular traits of fine roots (<2 mm in diameter), and their indicative value for recognizing strategies of soil resource exploitation and belowground competitive ability (Bauhus and Messier, 1999). Besides total root biomass and maximum rooting depth (Schenk and Jackson, 2002), important fine root morphological traits are specific root area (SRA, root surface area per mass), specific root length (SRL, root length per mass), root tissue density (RTD, mass per root volume), and fine root tip abundance (no. of tips per root mass) which may have a large influence on the rates of resource uptake (Jackson et al., 1997), root respiration (Pregitzer et al., 1998; Reich et al., 1998b) and rhizodeposition (Nadelhoffer and Raich, 1992; Jackson et al., 1997). Other functionally important traits are root N concentration and fine root lifespan that influence a root's economy of resource capture (Ryan et al., 1996; Pregitzer et al., 1998; Volder et al., 2005). Roots with greater length and surface development per biomass (high SRL and SRA) can explore larger soil volumes more efficiently and typically have higher resource uptake rates per unit root mass produced than roots with lower SRL and/or SRA. A higher surface (or length) per mass can be achieved either by reducing RTD or/and by decreasing root diameters (Eissenstat, 1991; Reich et al., 1998a; Ryser, 1998; Wright and Westoby, 1999). It has been found that root life span increases with growing RTD, decreased SRA and lowered root N concentration (Withington et al., 2006) in a similar manner as it is characteristic for leaf life span, specific leaf area (SLA) and foliar N concentration.

Despite their small contribution to overall tree biomass (Vogt et al., 1995), fine roots are functionally highly important tree organs that form the plant's interface with the soil and thus may sensitively reflect belowground responses to the environment. While basic knowledge exists about tree species differences in the structure and dynamics of fine roots (Leuschner and Hertel, 2003; Withington et al., 2006), root traits might also differ among the different genotypes of a species. However, information on the genetic background of intraspecific variation in fine root system structure and its architectural, morphological, and physiological properties is very scarce. This is also true for the linkage between root and leaf traits within the genotypes of a species (Ryser and Eek, 2000). A notable exception with respect to woody plants is the study by Withington et al. (2006) who compared various root traits among 11 temperate tree species and investigated root-shoot relationships on the species level.

The maintenance of intraspecific diversity (i.e., genetic diversity) is an important component of adaptive evolution, driving the ability of plants to colonize habitats of wide ecological amplitudes and to tolerate environmental change (Gregorius and Kleinschmit, 1999; Albert et al., 2011). Early-successional tree species such as *Betula, Populus*, and *Salix* taxa with broad ecological niches and large distribution ranges should reveal a particularly large intraspecific diversity with respect to leaf and root traits. Trembling aspen with the European species *Populus tremula* L. (European Common Aspen) and its close North American relative *Populus tremuloides* Michx. (American Quaking Aspen) belong to the most widespread woody species in the world (Hultén, 1986; Dickmann and Kuzovkina, 2008). Due to their large genotypic and also phenotypic variability, aspen may achieve a higher adaptability to future climatic changes than species with less intraspecific variation in leaf and root traits (Hamrick, 2004). Examining this variability particularly for root-related functional traits should substantially improve our understanding of the potential of trees to respond to different environmental conditions.

The present study investigates genotypic variation in fine root traits of aspen populations that originate from a broad range of sites in Central Europe and eastern North America with different climatic conditions. Aspen (*P. tremula* and *P. tremuloides*) as fast-growing pioneer trees with considerable drought tolerance and relatively low nutrient demand are one of the species being considered in short-rotation forestry for producing fiber, wood, and energy (Bradshaw et al., 2000; Taylor, 2002). Due to the continent-wide distribution, aspen may represent a promising study object for investigating genotypic and phenotypic variation in root traits and their linkage to variation in leaf traits. In plantation forestry, it is increasingly important to select genotypes which combine maximum wood production with broad tolerance of stresses associated with climate change. While the intraspecific variation in aboveground morphological, phenological, and physiological traits in aspen and their relation to growth have been investigated in much detail (e.g., Barnes, 1975; Calagari et al., 2006; Müller et al., 2012a), it is not known whether this variation is similarly reflected in root morphology. However, a better understanding of intraspecific trait variation in the root system and its dependence on the genetic relatedness between demes could improve the match between sown varieties and their growth conditions, hence improve growth performance under altered environmental conditions.

The overall goal of this study was to investigate how aspen demes of different geographic origin vary in important root and leaf morphological traits and biomass N contents when grown at a common site. Following the definition of Gilmour and Gregor (1939), we use the term “deme”, i.e., an assemblage of taxonomically closely related individuals, for identifying the progeny arrays. These poplar demes do not necessarily represent a specific taxonomic category (e.g., species, subspecies or varieties) or a specific origin of a species in the sense of a locally interbreeding population (Zhang, 2012). More specifically, we aimed to examine whether (1) the within-deme and between-deme variation in leaf morphological traits matches with similar patterns in root morphological trait variation, (2) the intraspecific variation in root and leaf morphology is related to genetic differences between the demes, and (3) how root and leaf morphological traits relate to aboveground productivity.

## Materials and methods

### Study site description

The study was conducted in the framework of the multidisciplinary experiment POPDIV at the University of Göttingen which investigates the role of intraspecific diversity in aspen for productivity and selected ecosystem functions. The common garden experiment was established on the Relliehausen Experimental Farm near Silberborn (51° 44′56″N, 09°32′28″E) in the Solling Mountains, about 60 km west of Göttingen (Lower Saxony, Germany). The study area is located at 485 m a.s.l. in the uplands of Central Germany with a sub-oceanic, cool-temperate climate (mean annual temperature of 6.6°C; annual mean precipitation of 1110 mm). The soil is unfertilized relatively nutrient-poor haplic Cambisol on Triassic sandstone (Middle Bunter) of sandy-loamy texture (Keuter et al., 2013). The site was previously used as extensive cattle pasture. A coring campaign prior to the experiment's start showed that the soil is homogenous across the site, thus effects of soil heterogeneity can be excluded throughout all 14 investigated blocks. Some soil characteristics are given in Table 1.

**Table 1:** **Soil characteristics of the experimental site (0–10 cm, total contents)**.

soil pH (H_2_O)	5.32 ± 0.21
C (%)	4.36 ± 0.03
N (%)	0.33 ± 0.01
K (mg g^−1^)	3.70 ± 0.02
Ca (mg g^−1^)	1.58 ± 0.02
Mg (mg g^−1^)	1.52 ± 0.01
Mn (mg g^−1^)	0.67 ± 0.01
Fe (mg g^−1^)	12.01 ± 0.08

Given are means ± se across 14 blocks (after Kleemann, 2010).

### Plant material

For the study, we used saplings of seven demes of *P. tremula* and one deme of the closely related *P. tremuloides*. The American taxon *P. tremuloides* and its close Eurasian relative *P. tremula* are either considered as sister species (Cervera et al., 2005; Pakull et al., 2009; Grant and Mitton, 2010) or as conspecific subspecies (Eckenwalder, 1996), depending on the criteria of relatedness used. Both taxa are assumed to have split in the late Miocene about 5–10 Ma ago (Schoell et al., 1994; Shevenell et al., 2004). The data on genetic differentiation among the demes, i.e., the analysis of simple sequence repeats (SSR) and amplified fragment length polymorphism (AFLP) markers, was kindly provided by the Department of Forest Genetics and Forest Tree Breeding at the University of Göttingen (Zhang, 2012). The places of origin cover a broad range of moderately warm to cool and oceanic to continental temperate climates and include gradients in mean annual temperature (5.4–10.7°C) and annual precipitation (592–1112 mm, Table 2). Saplings were raised from seeds or provided as wildlings and out-planted according to a randomized block design comprising 20 blocks (18.0 × 25.5 m) each consisting of six plots. All blocks were surrounded by an additional single tree row serving as buffer zone to avoid edge effects. In each plot, 25 3-y-old poplar plants of a deme were arranged in a rectangular grid with a plant distance of 1.5 m.

**Table 2:** **The eight aspen demes used in the study and their origin**.

**Acronym**	**Country, location**	**Type of culture**	**Coordinates**	**Elevation (m)**	**Mean annual precipitation (mm)**	**Mean annual temperature (°C)**	**Climate characteristics**
AU	Austria, Vienna	Seeds	48°16′N 16°19′E	390	600	9.9	Moderately cold winters, warm summers
CH	Switzerland, Birmensdorf	Seeds	47°21′N 08°24′E	692	1101	8.5	Moderately cold winters, moder. warm summers
G1	Germany, Ahrensbök	Seeds	53°59′N 10°38′E	25	664	8.8	Maritime winters, mild summers
G2	Germany, Göttingen	Seeds	51°32′N 09°56′E	315	645	8.7	Mild winters, moder. warm summers
G8	Germany, Göttingen	Seedlings	51°32′N 09°56′E	315	645	8.7	Mild winters, moder. warm summers
PL	Poland, Bialystok	Seedlings	53°08′N 23°09′E	160	592	6.7	Cold winters, moder. warm summers
S	Sweden, Edsvalla	Wildlings	59°26′N 13°12′E	101	635	5.4	Cold winters, cool summers
USA	U.S.A.: Mass., Boston/Sandwich	Seeds	42°14′N 71°23′W	80	1112	10.7	Relatively cold winters, warm summers

### Root collection and root trait analysis

For the root study, 44 of the 120 plots (in 14 of the 20 blocks) were chosen by random. Between June and early September 2010, fine root (<2 mm in diameter) samples were collected from 18–20 tree individuals per deme in the 44 plots; the sampled individuals were chosen by random from the each 25 plants per plot. With a spade, root samples were collected from the upper 30 cm of the mineral soil at a stem distance of 15–30 cm. To ensure that the root samples taken consisted indeed of fine roots of the nearby target tree, coarse roots from the respective stem were traced toward the terminal root endings and root coring was carried out at this location. We excavated soil monoliths of ~4000 cm^3^ volume containing coarse and fine roots of the respective plant individual, transported them to the laboratory and cleaned it with tap water from adherent soil. Fine roots of herbaceous plants were separated from the aspen fine roots and discarded. One aspen fine root branch of ~10 cm length was extracted from each monolith and used for subsequent analyses of root morphological traits and C and N concentrations in the dry mass. Thus, 18–20 replicate root samples per deme were analysed.

The fine root branches were spread out in a water bath and scanned for their surface area with a transmitting scanner system (Epson Expression 1680 1.0, Japan). Image analysis for determining the surface area, length and mean diameter of the root segments with a maximum diameter of 2 mm was conducted with WinRhizo software (Régent, Quebec, Canada). Additionally, the number of root tips per fine root individual was counted under a stereo-microscope and related to root dry mass. The analysed rootlets were oven-dried at 70°C for 48 h until constant weight. SRL (cm g^−1^) was calculated from root length divided by dry mass, SRA (cm^2^ g^−1^) and RTD (g cm^−3^) were obtained from surface area divided by dry mass or dry mass divided by fine root volume, respectively. The dried root material was ground and the C and N concentrations determined with an elemental analyser (Vario III EL, elementar Analysensysteme GmbH, Hanau, Germany).

### Leaf collection and leaf trait analysis

Simultaneously with root sampling, leaf samples were collected from the same 18–20 individuals per deme chosen for root sampling. Four leaves of the first-order twig on the main terminal shoot of a plant were collected from each tree. Digital images of the leaves were taken using a flatbed scanner (Epson Expression 1680 1.0, Japan). The images were analysed with the software WinFolia 2005b (Régent, Quebec, Canada) for their leaf area. The leaves were dried until constant weight at 70°C for 48 h and SLA calculated. The leaves were ground and the leaf material analysed for the C and N contents with an elemental analyser (Vario III EL elementar Analysensysteme GmbH, Hanau, Germany).

### Relative growth rate and aboveground biomass

Because the poplar plants were part of a long-term experiment, destructive harvests for determining biomass data and relative growth rate (RGR) directly were not possible. Alternatively, we estimated aboveground biomass (AGB) from root collar diameter (D_0_) and tree height (h) applying an allometric equation (Equation 1) established empirically by Heinrichs (2010) in a nearby young *P. tremula* stand on a forest clear-cut with similar site conditions.

(1) $$\text{AGB}=0.038\times {\text{D}}_{0}^{1.270}\times {\text{h}}^{1.388}$$

The calculation of aboveground productivity and aboveground RGR (g g^−1^ d^−1^) based on two sequential measurements of tree height and root collar diameter done for 4–15 plants per deme in April 2010 and April 2011.

### Statistical analyses

Statistical analyses were carried out with the software R, version 2.13.2 (R Development Core Team, 2011). The dataset was tested for normal distribution by the Shapiro & Wilk test. In case of non-gaussian distribution, the parameters were log-transformed to meet the assumptions of parametric tests. To test for heteroscedasticity, the fitted values were plotted against the residuals and inspected graphically. We used one-way ANOVA to analyse the influence of deme identity on the investigated morphological trait interactions. The General Linear Hypotheses (glht) procedure with Tukey's *post-hoc* test (contained in the “multcomp” -package) was applied to detect significant differences in the analysed trait means among the eight demes. Pearson correlation analysis was used to test for relationships between different root traits of the plants and for investigating inter-relationships between above- and belowground traits. To test for the relatedness of morphological trait variation and genetic variation across the eight demes, a Mantel test was performed (5000 permuted data sets) using the software Past (Hammer Harper and Ryan, 2001). The information on genetic differentiation among the demes, which bases on the analysis of SSR and AFLP markers, was kindly provided by the Department of Forest Genetics and Forest Tree Breeding at the University of Göttingen (Zhang, 2012). We calculated coefficients of within-deme variation (CV_intra_) and of between-deme variation (CV_inter_) using Equation 2:

(2) $${\text{CV}}_{\left(\text{in percent}\right)}=\text{SD}/\text{mean}\times 100$$

for allocating total measured trait variation to a genetic component (CV_inter_) and a predominantly environmental component (CV_intra_).

A Principal Components Analysis (PCA) was performed on leaf and root morphological and growth-related traits using the software Canoco for Windows 4.5. Means of all investigated parameters were standardized and constructed on the two main axes (PC1 and PC2) in the orthogonal plane in addition to the allocation of the eight demes.

All traits that were found in the PCA to be most closely related to RGR were used as explanatory variables in a multiple linear regression to identify their relative importance for plant productivity; traits with close interrelationship or derived from each other were excluded (except for leaf size and SLA). Multiple linear regressions were calculated by stepwise backward model selection using the “stepAIC”-function from the “MASS”-package (Venables and Ripley, 2002) for model simplification.

## Results

### Between- and within-deme variation in root morphology and root N concentration

Five of the six investigated root traits (diameter, SRA, SRL, root tip abundance, root N concentration) differed significantly between the demes while one (RTD) did not (Table 3). Mean fine root diameter was very uniform across the seven *P. tremula* demes (means: 0.23–0.27 mm), while the American *P. tremuloides* deme had a significantly larger mean diameter (0.30 mm; Table 4). The relatively large diameter of this deme corresponded to a particularly small SRA and SRL, while the G1, G2 and G8 demes (*P. tremula*) had the highest SRA and SRL means in correspondence with low diameters (0.23–0.25 mm; however, the difference between these two deme groups mark only a non-significant trend Table 4). The highest between-deme variation was observed for root tip abundance (means ranging from 22.5–39.1 n mg^−1^; between-deme variation 47.7%; Table 4). The root N concentration mean ranged between 1.39 and 1.75% among the demes and between-deme variation was relatively small (21.3%). RTD was not significantly different between the demes (Table 4). The three demes Austria (AU), Germany (G1) and Poland (PL) showed a particularly high within-deme variation that exceeded for most of the seven root traits the between-deme variation. In the other five demes, CV_intra_ was mostly smaller than CV_inter_. Between-deme (genetically-determined) variation was largest in root tip abundance and SRL, intermediate in SRA and root N concentration, and lowest in root diameter (Table 4).

**Table 3:** **Results of ANOVA on leaf and root trait differences between the eight demes**.

**Trait**	***F***	***df***	***P***
Relative growth rate	4.34	73	**<0.001**
Leaf size	29.80	146	**<0.001**
SLA	3.49	146	**<0.001**
Leaf N concentration	3.26	146	**<0.01**
Fine root diameter	5.80	146	**<0.001**
SRA	2.73	146	**<0.01**
SRL	3.84	146	**<0.001**
Tip abundance	5.33	145	**<0.001**
RTD	2.04	146	n.s.
Root N concentration	3.25	146	**<0.01**

Parameters with significant variation across the demes are printed in bold.

**Table 4:** **Morphological and chemical traits of leaves (fully expanded leaves on terminal twigs) and fine roots (<2 mm in diameter) of the eight aspen demes and their mean relative growth rates (RGR, only aboveground biomass; in mg g^−1^d^−1^ for the period April 2010 to April 2011)**.

	**AU**	**CV intra (%)**	**CH**	**CV intra (%)**	**G1**	**CV**	**G2**	**CV intra (%)**	**G8**	**CV intra (%)**	**PL**	**CV intra (%)**	**S**	**CV intra (%)**	**USA**	**CV intra (%)**	**CV intra (%)**
RGR (mg g^−1^d^−1^)	3.20 ± 0.55ab	48.44	2.46 ± 0.42b	64.5	3.67 ± 0.68ab	42.7	1.68 ± 0.24b		2.42 ± 0.96b	88.1	1.59 ± 0.90ab	−	5.25 ± 0.69a	49.2	5.55 ± 0.98a	43.15	76.55
**LEAF TRAITS**																	
Leaf size (cm^2^)	10.24 ± 0.84a	36.6	6.4 ± 1.02b	69.7	5.55 ± 0.50b	39.6	5.15 ± 0.40b	34.5	4.68 ± 0.50b	48.3	8.19 ± 0.93ab	48.0	6.31 ± 0.63b	44.3	19.11 ± 1.58c	35.2	70.5
SLA (cm^2^ g^−1^)	105.48 ± 3.25ab	13.8	102.68 ± 2.57b	10.9	119.51 ± 5.19a	18.9	102.96 ± 2.56b	11.1	109.47 ± 3.31ab	13.5	111.33 ± 3.62ab	13.8	110.15 ± 2.23ab	9.0	100.05 ± 3.03b	12.9	14.2
Leaf N (%)	2.65 ± 0.09a	15.9	2.45 ± 0.11ab	19.5	2.52 ± 0.07ab	12.1	2.22 ± 0.06b	12.8	2.24 ± 0.10b	20.0	2.41 ± 0.10ab	18.1	2.21 ± 0.08b	15.4	2.42 ± 0.07ab	12.6	16.9
**ROOT TRAITS**																	
Diameter (mm)	0.27 ± 0.01ab	11.7	0.25 ± 0.01bc	9.5	0.23 ± 0.01c	16.4	0.25 ± 0.01bc	8.9	0.25 ± 0.01bc	9.2	0.26 ± 0.01bc	18.7	0.26 ± 0.01bc	15.7	0.30 ± 0.01a	18.9	15.8
SRA (cm^2^ g^−1^)	392.77 ± 31.83a	36.2	455.13 ± 22.28ab	21.3	501.09 ± 35.21ab	30.6	515.73 ± 27.28ab	23.7	530.91 ± 23.95b	20.2	454.80 ± 37.61ab	35.1	471.07 ± 29.01ab	27.5	415.64 ± 23.78ab	24.3	28.5
SRL (m g^−1^)	58.65 ± 6.10a	46.5	74.74 ± 4.60ab	26.8	90.59 ± 8.97b	43.2	84.83 ± 5.99ab	31.6	88.82 ± 4.72b	23.7	74.73 ± 8.22ab	46.6	75.14 ± 5.31ab	31.6	58.61 ± 5.31a	38.4	38.6
RTA (n mg^−1^)	22.48 ± 2.47ab	49.2	30.37 ± 2.28bc	32.8	39.09 ± 3.68c	41.0	33.83 ± 2.46ac	32.5	36.08 ± 2.56c	31.0	31.13 ± 3.43bc	46.7	33.3 ± 2.55ac	34.2	20.58 ± 2.37b	48.8	42.7
RTD (g cm^−3^)	0.31 ± 0.01a	n.s.	0.32 ± 0.02a	n.s.	0.34 ± 0.02a	n.s.	0.28 ± 0.01a	n.s.	0.29 ± 0.01a	n.s.	0.31 ± 0.02a	n.s.	0.31 ± 0.02a	n.s.	0.27 ± 0.01a	n.s.	n.s.
Root N (%)	1.45 ± 0.07ab	21.1	1.73 ± 0.07b	18.1	1.54 ± 0.09ab	24.7	1.68 ± 0.06ab	16.6	1.75 ± 0.07b	19.2	1.39 ± 0.07a	21.0	1.52 ± 0.06ab	16.3	1.53 ± 0.09ab	25.4	21.3

*For deme acronyms see Table 2. The eight aspen demes used in the study and their origin. Given are means ± se, morphological and chemical traits: n = 18 − 20, relative growth rate n = 4 − 15; p < 0.05. The coefficient of variation within the demes (CV_intra_, %) and between the eight demes (CV_inter_, %; last column) are given. Different letters indicate significant differences in the means between the demes. RTA − root tip abundance*.

### Between- and within-deme variation in leaf morphological and chemical traits

Leaf N concentration showed a similarly small variation between the demes (means of 2.21–2.65%; CV_inter_ = 16.9%) as root N concentration. A low between-deme variation (14.2%) was also found for SLA (relatively high SLA means in the *P. tremula* deme G1, particularly low SLA in the *P. tremuloides* deme USA; Table 4). In contrast, leaf size was the trait with by far largest between-deme variability (70.5%; Table 4). The *P. tremuloides* deme had a four times greater mean leaf size than the deme with smallest leaves (G8) and it exceeded the deme with second largest leaves (AU) nearly twofold (Table 4). In contrast to all other investigated leaf or root traits, leaf size showed a much smaller within-deme than between-deme variation (34.5–69.7 vs. 70.5%). A larger leaf size was associated with a higher foliar N concentration; leaf N also increased with increases in SLA (Table 5). SLA itself was not related to leaf size in our sample.

**Table 5:** **Pearson correlation coefficients for linear relationships between three leaf and six root traits across the eight demes (*n* = 154)**.

	**Leaf size**	**SLA**	**Leaf N**	**Root diam.**	**SRA**	**SRL**	**Tip abund.**	**RTD**	**Root N**
Leaf size	-								
SLA	−0.11	-							
Leaf N	0.24^**^	0.32^***^	-						
Root diameter	0.22^**^	−0.23^**^	−0.02	-					
SRA	−0.18^*^	0.09	−0.03	−0.52^***^	-				
SRL	−0.21^**^	0.15	−0.01	−0.70^***^	0.95^***^	-			
Tip abundance	−0.26^**^	0.15	−0.02	−0.63^***^	0.80^***^	0.84^***^	-		
RTD	−0.06	0.07	0.07	−0.27^***^	−0.54^***^	−0.29^***^	−0.22^**^	-	
Root N	−0.12	0.01	0.10	−0.29^***^	0.46^***^	0.46^***^	0.36^***^	−0.13	-

Significant correlations are marked by

* (p < 0.05),

** (p < 0.01) or

*** (p < 0.001) and are printed in bold.

### The influence of genetic variation on leaf and root trait variation

The results of the Mantel test revealed a close relation between the genetic variation among the demes as visible in the AFLP markers and the variation in aboveground plant biomass recorded for the demes in the year 2011 (*r* = 0.87, *p* = 0.04). Significant relations were also observed for the parameters leaf size and SLA, whereas aboveground growth rate (RGR) and leaf N concentration revealed no correspondence in the distances between the molecular and the trait datasets (Table 6). From all investigated root traits, only RTD and root tip abundance showed a significant correspondence between the two data matrices, while the other root traits (root diameter, SRA, SRL, root N concentration) varied independently from genetic variation across the demes. None of the morphological parameters revealed significant relations to SSR markers (data not shown). When the leaf or root traits are pooled in the Mantel test analysis, e.g., all investigated leaf traits or all root traits were merged together, the relations remained significant for the aboveground parameters (*r* = 0.77, *p* = 0.001), while this was not the case for the root traits (*r* = 0.71, *p* = 0.07). When all measured above- and belowground traits were investigated together, the relation was significant (*r* = 0.84, *p* = 0.05).

**Table 6:** **Results of a Mantel test conducted to analyse the relationship between morphological trait variance (first matrix) and genetic variance according to AFLP markers (second matrix) in the sample of eight demes**.

	**Mantel's *r***	**Probability *P***
Aboveground RGR	0.416	0.082
Aboveground biomass 2010	0.310	**0.025**
Aboveground biomass 2011	0.870	**0.041**
Leaf size	0.916	**0.040**
SLA	0.362	**0.002**
Leaf N concentration	−0.165	0.773
Fine root diameter	0.855	0.087
SRA	0.280	0.196
SRL	0.478	0.065
Root tip abundance	0.493	**0.047**
RTD	0.518	**0.046**
Root N concentration	0.516	0.129
All leaf morphological traits	0.767	**0.009**
Biomass and growth traits	0.784	0.055
Leaf morphological traits	0.852	**0.009**
All root traits	0.711	0.074
All root morphological traits	0.567	0.067
All traits	0.840	**0.047**

Significantly correlating leaf or root traits are printed in bold (*p* < *0.05*).

### Relationships among leaf traits, root traits and RGR

As expected, SRA and SRL showed highly significant negative correlations to root diameter across the sample (*p* < 0.001, *r* = −0.52 and −0.70, respectively; Table 5). Further, we found inverse relations of RTD to SRA, SRL, and root diameter, i.e., higher tissue densities in thinner roots. Root tip abundance increased with SRA and SRL and decreased with increasing diameter (Table 5). Roots with smaller diameter but relatively high SRA and SRL had higher root N concentrations; low tissue density was also linked to higher N concentrations.

Of the 18 tested relationships between root and leaf traits, only five were significant. Demes with higher SLA had smaller fine root diameters, and large-leaved demes had larger root diameters but lower SRA, SRL and tip numbers than demes with smaller leaves (Table 5). No significant relationships were found between leaf N concentration and root N concentration, and between SLA and SRA or SRL. While root N concentration and leaf N concentration showed similar variation among the eight demes (CV_inter_ values of 21.3 and 16.9%), SRA and SRL were more variable than SLA (CV_inter_: 28.5 and 38.6% vs. 14.2%). Mean leaf size varied up to twofold among the demes and showed a higher total variation (CV_inter_: 70.5%) than any root trait.

**Figure 1:**
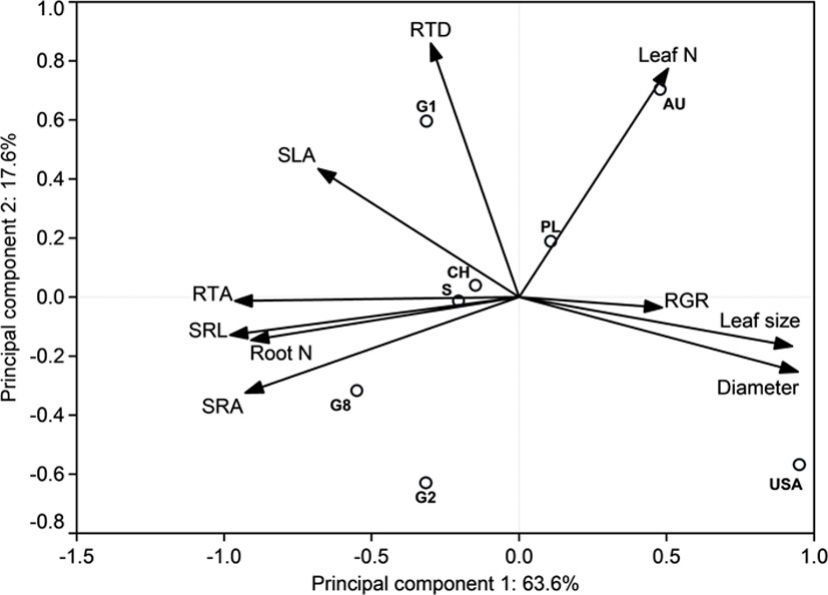
**Distribution of relative growth rate and root and leaf morphological properties in the orthogonal plane of the Principal Components Analysis for the eight poplar demes and the percentage contribution of the respective axis to total variability, *n* = 4–15 individuals per deme**. G1, G2, G8, CH, S, PL, AU and USA stand for the eight demes.

The estimate of mean aboveground RGR for the period April 2010 to April 2011 revealed large differences between the eight demes. The demes with highest RGR (USA: 5.55 and S: 5.25 mg g^−1^ d^−1^) grew more than three times faster than the two demes with lowest growth rate (PL: 1.59 and G2: 1.68 mg g^−1^ d^−1^) (Table 4). The other four demes reached intermediate rates (2.42–3.67 mg g^−1^ d^−1^). The two main axes of the PCA explained 81% of the variability in the ten investigated above- and belowground variables including RGR (Table 7, Figure 1). Axis 1 with an eigenvalue of 0.64 was strongly positively correlated with leaf size and fine root diameter but negatively with the fine root morphological traits SRA, SRL, the number of root tips and root N concentration. However, none of these root traits were significantly related to RGR indicating that the studied aspen genotypes do not achieve faster aboveground growth through alteration of root morphological characteristics in the range of trait variability investigated here. The second axis (eigenvalue 0.18) was primarily associated with leaf N concentration and RTD. Axis 3 contributed with only 11% to the variance and was strongly related to RGR, with no other trait being significantly related to this axis.

**Table 7:** **Principal Components Analysis of the eight poplar demes with respect to relative growth rate and leaf and root morphological properties**.

**Variables**	**Axis 1**	**Axis 2**	**Axis 3**
	EV (0.636)	EV (0.176)	EV (0.105)
**GROWTH-RELATED VARIABLE**			
Aboveground RGR	0.484	−0.035	**0.858**
**LEAF-RELATED VARIABLES**			
Leaf size	**0.925**	−0.167	0.264
SLA	−**0.680**	0.434	0.364
Leaf N concentration	0.504	**0.773**	−0.159
**ROOT-RELATED VARIABLES**			
Fine root diameter	**0.944**	−0.253	−0.018
SRA	−**0.927**	−0.324	0.102
SRL	−**0.979**	−0.129	0.103
Root tip abundance	−**0.963**	−0.013	0.212
RTD	−0.299	**0.859**	0.077
Root N concentration	−**0.908**	−0.146	−0.120

*Given are the loadings of the selected variables along the three most important explanatory axes. Eigenvalues are given in brackets in the headline. Numbers in bold mark the variables with the closest correlation to the respective axis (n* = 4 – 15 *individuals per deme)*.

A multiple regression analysis with backward variable selection of the possible growth-influencing factors leaf size, SLA, SRL and root tip abundance as predictor variables identified none of the belowground traits as influencing RGR, while leaf size (as a proxy of total leaf area) was detected as the single most important trait. However, the model fitted for the whole data set (eight demes) explained only 18% of the RGR variation (data not shown).

## Discussion

### The aspen fine root system: genotypic variation vs. phenotypic plasticity

Across the eight demes and the 18–20 plants investigated per deme, fine root morphology showed a high variability in all parameters except in fine root diameter. Despite identical climatic conditions and uniform soil across the experimental site, within-deme variation was considerable which may be explained either by genetic variation within the deme or by small-scale soil heterogeneity (e.g., variable stone content at the plant scale). The 18–20 plants of a deme varied in their genetic constitution to a certain degree because they were reared after natural pollination on the same parent tree or represent the offspring of a few trees of a population. This genetic variation should add to the phenotypic plasticity due to small-scale environmental variation at the experimental site. An experiment with clonal plants instead of plants reared from seed would allow differentiating between the effects of genetic variability and those of phenotypic plasticity on root morphology. Measuring errors are another likely source of variation. The remarkably small variation in root diameter found across the ~160 aspen plants has to be interpreted with care. It is well recognized that mean fine root diameter is not a good descriptor for the large variation in root morphology and function occurring along the branching hierarchy from the root ending to higher root orders (Pregitzer et al., 2002; Goebel et al., 2010; Rewald et al., 2011; Beyer et al., 2013). Inherent trait variation within the fine root system has also been found in other root traits and it should determine the uptake capacity for water and nutrients through alteration in root surface area or specific root length. For example, even though the means of SRL and root N concentration were similar to our data, these traits varied by a factor of two among the different fine root orders in the *Populus balsamifera* plants examined by Pregitzer et al. (2002). A more detailed analysis of aspen root systems based on root orders might well have detected morphological differences between the demes that were not visible in our analysis. All five investigated root morphological traits revealed a within-deme variation that was in the same magnitude or higher than between-deme variation. Addressing our second study objective, these findings indicate that the studied traits do not underlie strong genetic control. High phenotypic plasticity represents an adaptive advantage when resource availability varies rapidly in time and space as is the case in soils where alternating periods of infiltration and soil drying and pulsed nutrient release from mineralization require a high flexibility in the placing of roots and in root uptake activity. In contrast to root morphological traits, genotype had a strong influence on leaf morphology and aboveground plant biomass what is in line with a former study by Müller et al. (2012a).

Highly variable environmental conditions such as N and water availability exert a large influence on the structure and morphology of plant root systems; this may often mask the genotypic influence (e.g., Lohmus et al., 1989; Ostonen et al., 2007). Strategies for capturing belowground resources at minimal costs include the production of fine roots with high SRL and SRA allowing to achieve high root length densities in large parts of the soil at relatively low cost, or growing roots selectively into nutrient hotspots and moist patches as observed in two grass species (Mommer et al., 2011). *Populus* species produce very thin roots and can reach much higher SRL than other North American tree species (Pregitzer et al., 2002), what is in line with the observed fast spread of the mainly lateral-distributed root systems of poplars (Pregitzer and Friend, 1996). Intensive lateral root growth indicates that poplars seem to follow strategies of short-term reaction to nutrient hotspots rather than maintaining active root systems in large soil volumes. Such a strategy would fit the adaptation to unstable habitats such as bare sandy soils or flooded alluvial soils where many poplars thrive.

### Co-variation between root and leaf traits

In grassland plants, quite a number of studies have examined the interrelation between leaf and root traits for characterizing resource economic trade-offs, mostly with a focus on SLA and SRA or SRL, or leaf and root N concentrations (e.g., Craine and Lee, 2003; Craine et al., 2005; Tjoelker et al., 2005). As far as we know, our study is the first to search for co-variation in leaf and root traits among different genotypes of a single tree species or species aggregate. Across the eight aspen demes, SLA was inversely correlated with fine root diameter in a similar manner as it was found by Withington et al. (2006) in 11 Central European tree species. In contrast, the SLA–SRL relation was not significant in our study, even though we investigated a total of ~160 plants. The missing SLA–SRL relation in aspen matches with results obtained from the comparison of different grass species (Reich et al., 2003; Tjoelker et al., 2005), but contrasts the tighter SLA–SRL relation detected when comparing the seedlings of different tree species (Reich et al., 1998a; Wright and Westoby, 1999; Withington et al., 2006). A significant relation between root and leaf N concentrations was also lacking in our aspen deme sample which contrasts with the close inter-relationships detected in grass species by Craine and Lee (2003), Craine et al. (2005), Tjoelker et al. (2005) but is in accordance with findings from 11 temperate tree species by Comas and Eissenstat (2009). We also tested for deme differences in the relationship between root and shoot traits using linear models with deme and the respective root trait as explanatory factors and SLA or leaf size as dependent variables, but similarly did not find a significant deme effect on the root-leaf trait linkage. It appears that the significance of inter-relationships between leaf and root properties in a plant is dependent on the variation in plant architectural types and life forms covered by the analysis. The range of trait variation is typically smaller in intraspecific than interspecific comparisons (Comas and Eissenstat, 2009) with the consequence that possible relationships between root and leaf traits may well be masked when the within-deme variation in root traits is high as in our study. Again, a root order-related analysis of root traits might have revealed clearer relations between root and leaf traits even at the intraspecific level. However, applying a more sophisticated root order-related approach would result in a reduced number of replicate root samples that can be processed in due time.

### Root trait variation and plant growth

Only few studies have examined how root traits are related to plant productivity and growth strategies. Most of the relevant research was carried out with tree seedlings (Reich et al., 1998b; Wright and Westoby, 1999; Comas et al., 2002) or herbaceous plants in greenhouse experiments. Comas and Eissenstat (2004) studied the relation between fine root morphology and chemistry, and growth rate in six-year-old fast- or slow-growing deciduous tree species and found that trees with high potential growth rates constructed roots with smaller diameter, higher SRL, more root tips per unit length and higher root N concentration. In contrast, the recent results of Tobner et al. (2013) did not confirm significant coordination of fine root traits and RGR across North American temperate tree species. Observations in our study hint to the better studied aboveground trait syndromes where high RGR is typically associated with high SLA (Poorter and Garnier, 1999) and a high leaf mass ratio (leaf mass per plant mass) (Poorter and Remkes, 1990; Walters et al., 1993), high shoot N contents and a relatively short leaf longevity (Wright and Westoby, 2000). Müller et al. (2012a,b) conducted a detailed growth analysis in four of the eight aspen demes of this study searching for growth-determining leaf and shoot traits. They concluded that aboveground RGR was primarily determined by total leaf area which itself was largely dependent on the onset of leaf abscission in early autumn in the aspen plants with their continuous leaf production throughout the growing season. Leaf assimilation rate was of minor importance; root traits were not investigated. The results of our regression analysis, which included the aboveground variables leaf size (as a proxy of total leaf area) and SLA and the belowground parameters SRL and root tip abundance, also showed leaf size to be the principal determinant of RGR in the eight-deme sample. Both the PCA and the multiple regression analysis revealed that root traits in general had only a weak or even no influence on aboveground RGR.

We had assumed that the aspen demes with highest SRA and SRL would grow fastest because high growth rates are generally linked to high rates of water and nutrient consumption (Van den Driessche et al., 2003) requiring root systems with high uptake capacity as indicated in the study of Comas and Eissenstat (2004). Long thin roots with high SRL and SRA should be more effective in the exploration of water and nutrient reserves in a given soil volume (e.g., Bauhus and Messier, 1999). However, they may be more costly in terms of plant resources needed for building them as compared to roots with smaller surface per mass ratios because the former are typically turned over faster and often contain more N per dry mass (Reich et al., 1998b). Surprisingly, we found in the aspen demes a tendency for a negative relation between (aboveground) RGR and SRA, SRL, and root tip abundance, while growth rate seemed to increase with growing fine root diameter. Even though this relation was not significant, it suggests that these root characteristics are not important for aboveground productivity.

The lack of a linkage between fast growth and a high specific fine root surface area (and root traits in general) may have several reasons. First, we investigated only aboveground, but not belowground productivity. Rapid growth requires a high leaf mass ratio which could lead to simultaneous resource limitation for root growth, demanding for the production of less costly thicker roots with higher longevity. Second, fast-growing trees with higher demand for soil resources can achieve the required uptake capacity either by producing thinner more active fine roots, which explore the space more intensively, or by extending their root system if sufficient unexplored soil space is available. The three-year-old aspen plants were still in the stage of expansive root system growth when root sampling took place. Thus, it is possible that the fast-growing demes achieved the assumed higher uptake rate mainly through root system extension and not by forming thinner, more uptake-efficient roots. Unfortunately, we have no information on total root mass and root system size in the eight demes. Finally, genotypic differences in root growth phenology could be as influential, or even more important, for RGR than root morphological traits. Pregitzer and Friend (1996) showed that fast growth in young *Populus* trees was associated with early root growth. Müller et al. (2012a,b) identified phenological traits (the timing of bud burst and the onset of leaf abscission in late summer) as key factors determining aboveground productivity in *P. tremula*. While we found bud burst to differ by two weeks among the demes, we have no data on root phenology.

The aboveground phenological traits of aspen seem to be largely under genetic control but they showed no simple relation to the latitude or temperature at the place of origin (Kleemann, 2010; Müller et al., 2012a). Monitoring of root growth and death by direct observation techniques has to show whether root phenology is indeed a factor influencing aboveground productivity, and how it depends on genetic or environmental control.

## Conclusions

The fine root system of three-year-old aspen progenies (demes) from origins with broadly contrasting climate differed significantly in several morphological traits indicating that SRA, SRL, RTD, tip abundance and mean root diameter are at least to some extent determined by the genetic constitution. However, within-deme variation in the each 18–20 plants was of similar magnitude as between-deme variation, demonstrating a high intraspecific morphological plasticity of the fine root system probably in response to small-scale soil heterogeneity. We did not find a significant relationship between morphological trait variance and genetic variance suggesting that genetic distance is not an important determinant of root trait divergence. The relation between analogous above- and belowground traits was not very tight at the intraspecific level, probably due to masking by high within-deme variation. The large differences in aboveground RGR among the eight demes were tightly linked to genetically determined leaf morphological and phenological traits but were only to a small extent explained by variation in fine root morphology. Even though the studied fine root traits seem not to be good predictors of aspen growth performance, we need more information on genotypic differences in root morphology and function for aspen progenies and other fast-growing tree species used in short-rotation forestry. The limitations of a simple categorization of fine root biomass into diameter classes suggest applying a morphometric approach based on the separation of root orders for coping with the hierarchical heterogeneity in anatomy, chemistry and function of the branching structure of the fine root system. This may allow characterizing specific belowground resource acquisition and allocation strategies among different provenances of a tree species.

### Conflict of interest statement

The authors declare that the research was conducted in the absence of any commercial or financial relationships that could be construed as a potential conflict of interest.
